# Supplementary material for: Bi-allelic MCM10 variants associated with immune dysfunction and cardiomyopathy cause telomere shortening
Source: Nat Commun. 2021 Mar 12;12:1626. doi: 10.1038/s41467-021-21878-x (PMC7955084; doi:10.1038/s41467-021-21878-x)

SUPPLEMENTARY INFORMATION

Supplementary Note 1

Restrictive cardiomyopathy with lymphoreticular hypoplasia

The proband was a male fetus of a gravida 2 para 1 healthy Caucasian female. There was no familial consanguinity. A routine antenatal scan at 12 weeks gestation was normal. Routine antenatal scanning at 20 weeks gestation revealed a pericardial effusion. Follow up scans showed progressive fetal hydrops and reduced cardiac function, with cardiac morphology suggestive of a restrictive cardiomyopathy (RCM). Intra-uterine death was recorded at 27 weeks gestation and the fetus was delivered. Karyotyping on amniotic fluid was normal. A post mortem examination was undertaken. No dysmorphic facial features, limb abnormalities or major structural cardiac abnormalities were noted. However, the right atrium (RA) was severely dilated, and the left ventricle (LV) was thickened. Myocardial histology was non-specific but showed enlarged myofiber nuclear size and endocardial fibroelastosis consistent with a cardiomyopathy. The thymus and spleen were hypoplastic, weighing 0.8 g (normal range for gestation 2.04 +/- 0.39 g) and 0.82 g (normal range for gestation 1.24 +/- 0.36 g), respectively. Histology of the spleen showed autolyzed tissue with no specific pathological features. Further molecular genetic investigation including a 57 gene “cardiomyopathy panel”, mitochondrial genetic analysis and oligonucleotide array comparative genomic hybridization (CGH) revealed no plausible pathogenic variant.

The next pregnancy underwent detailed antenatal ultrasound scans at 9 and 12 weeks of gestation that were normal. A scan at 16 weeks revealed ascites with pleural

and pericardial effusions. Cardiac function was reduced. Termination of pregnancy was carried out at 17 weeks gestation. No dysmorphic features were noted. There were no limb abnormalities. The RA was severely dilated, and the LV was larger than the RV, but no structural congenital cardiac defect was identified. Myocardial histology revealed enlarged myofiber nuclei with endomyocardial fibroelastosis. The thymus and spleen were hypoplastic, weighing 0.04 g (normal range for gestation 0.18 +/- 0.06 g) and 0.05 g (normal range for gestation 0.14 +/- 0.07 g), respectively. Histology of both was unremarkable.

The third affected pregnancy followed a remarkably similar course to those described above with an antenatal diagnosis of RCM with fetal hydrops. Termination of pregnancy was undertaken. The RA was enlarged but no structural heart disease was noted. Myocardial histology revealed myofiber nuclear hypertrophy and endomyocardial fibroelastosis, consistent with a diagnosis of cardiomyopathy. The thymus and spleen were hypoplastic, weighing 0.32 g (normal range for gestation 1.71 +/- 0.54 g) and 0.69 g (normal range for gestation 1.05 +/- 0.25 g), respectively. The parents and three other clinically unaffected siblings had a normal ECG and echocardiogram.

Exome and genome sequencing of RCM patients and family

Exome sequencing of the parents and proband yielded no strong candidate variants from a panel of known cardiomyopathy associated genes. A gene agnostic trio analysis was undertaken. This generated an extensive list of variants of uncertain significance. To reduce the number of candidates and/or identify additional variants the third affected fetus underwent genome sequencing, and this data was analyzed together

with the original three exomes. Given the pedigree structure, analysis focused on autosomal recessive and X-linked modes of inheritance. Compound heterozygous variants c.[236delG];[764+5G>A] in *MCM10* (NM_018518.5; Fig. 1a) remained plausible candidate variants following this quad analysis. These variants were validated using Sanger sequencing and were shown to segregate within the family in a manner consistent with them being autosomal recessive pathogenic variants (Fig. 1b). Each unaffected individual had at least one wild type *MCM10* allele.

Supplementary figure legends

Supplementary Fig. 1. Identification and modeling of *MCM10* patient variants.

a) Proband sequencing analysis validating the c.236delG variant. Base calls are shown below the trace image with the wild type allele (top) and mutant allele (bottom) shown separately on the 3' end. The corresponding protein translation is shown below each trace image. (Right) Schematic of the wild type and mutant c.236delG exon 3 *MCM10* alleles.

b) Sequencing analysis of cDNA from the mother showing the wild type *MCM10* exon5/6 junction and from the father validating the c.764+5G>A variant that results in loss of exon 6 from the mature mRNA. The corresponding protein translation is shown below each trace image. Below each translation a bar indicates regions coded for by *MCM10* exon 5 (red), exon 6 (green) or exon 7 (blue). (Right) Schematic of the wild type and mutant c.764+5G>A alleles and the location of the exon 7 cryptic splice acceptor (cryptic SA). Annotations in a and b are in reference to *MCM10* transcript NM_018518.5.

c) Comparison of splice donor (light gray) and acceptor (dark gray) strength for each *MCM10* splice site indicated, normalized to a consensus splice site sequence using the MaxEntScan webtool[1]. Results using three different scoring methods are indicated as: 1) Maximum Entropy Model, 2) First-order Markov Model, or 3) Weight Matrix Model.

d) Adapted cartoon of human MCM10[2] including the N-terminal domain that harbors a coiled-coil (CC, orange) motif. The internal domain contains a PCNA-interacting peptide (PIP) box (red), Hsp10-like domain (purple), an oligonucleotide/oligosaccharide binding (OB)-fold (gray) and zinc-finger motif 1 (ZnF1, green). The C-terminal domain contains ZnF2 (green), the zinc ribbon (ZnR, blue) and winged helix motif (WH, light gray). MCM10 structural domains are connected by two flexible linker regions (yellow). The location of

the RCM-associated G79EfsTer6, D198GfsTer10, and potential Δ198-262, and NKD-associated R426C and R582X variants are indicated. Cartoon adapted by permission from Springer Nature: Springer International Publishing Switzerland, The Role of Mcm10 in Replication Initiation by Baxley R.M., Thu Y.M., and Bielinsky AK. In: The Initiation of DNA Replication in Eukaryotes, Kaplan D. (eds). Copyright 2016.

e) CD spectra comparing WT and ΔN27 MCM10-ID at 25˚C and 60˚C.

f) Melting curves for WT and ΔN27 MCM10-ID obtained by plotting the normalized CD signal (ellipticity) at 205 nm from 40˚C to 90˚C.

Supplementary Fig 2. Characterization of engineered mutations and cell death in *MCM10* mutant cell lines.

a) UCSC genome browser view of alignments of *MCM10* exon 14 Sanger sequencing reads from clones #8 and #14 using forward (FW) and reverse (RV) primers. For each sequence, correct alignment with the reference genome is indicated by black color. Incorrect alignment, if present, is indicated in red.

b) Alignment of each mutation carried by HCT116 or RPE-1 cell lines to the wild type *MCM10* sequence within exon 3 over the locus targeted by CRISPR-Cas9 to generate each cell line.

c) UCSC genome browser view of *MCM10* exon 3 indicating the location of each mutation carried by HCT116 or RPE-1 cell lines, as well as the RCM-associated patient variant.

d) Example flow cytometry gating for analysis of apoptosis in HCT116 and RPE-1 cells.

e) Representative flow cytometry plots indicating the average percentage of each population represented by early apoptotic, late apoptotic or dead cells as detected using

a combination of propidium iodide and annexin V staining of HCT116 wild type and *MCM10*$^{+/-}$ cell lines.

Supplementary Fig. 3. Characterization of genomic instability in *MCM10*$^{+/-}$ cell populations.

a) Representative phase contrast images of early, middle and late passage *MCM10*$^{+/-}$ cell populations. Scale bar is 125 μM.

b) Example karyotypes from late passage HCT116 wild type (top) and mid-passage (middle) or late passage (bottom) *MCM10*$^{+/-}$ cells. Blue arrows indicate expected HCT116 genomic aberrations. Red arrows indicate non-clonal genomic aberrations.

c) TRF analysis comparing early passage HCT116 wild type cells with clonal MCM10-deficient populations carrying inactivating mutations in one copy of *MCM10* exon 3. Location of peak intensity (yellow dots) is indicated.

d) Quantification of average β-gal activity expressed as arbitrary fluorescence units normalized to total protein for HCT116 wild type (blue) and clonal *MCM10*$^{+/-}$ cell lines (red). Error bars indicate SD and n = 3. Individual data points are indicated (gray circles).

e) Quantification of signal free-ends (left) and fragile telomeres (right) in late passage HCT116 wild type (blue) and *MCM10*$^{+/-}$ cells (red); n = number of metaphases quantified. Error bars in d and e indicate SD and significance was calculated using an unpaired, two-tailed student's *t-test* with *<.05; **<.01, ***<.001.

f) Genotyping of wild type (middle) or mutant alleles carrying a *loxP* site 3' of exon 14 (upper) or a *loxP* scar (lower). A faint non-specific band is noted (asterisk).

g) Flow chart for experiment to generate and analyze PURO-marked *MCM10*$^{+/-}$ cell lines.

h) Analysis of a PURO-marked HCT116 *MCM10*$^{+/-}$ #14 cell population tracking average telomere length by TRF (top) and reversion of the exon 14 locus by PCR (bottom). PDs and location of peak intensity (yellow dots) are indicated. Source data for panels **c**, **d**, **e**, **f** and **h**, including relevant exact p-values, are provided in the Source Data file.

i) Schematic depiction of the proposed mechanism(s) for genetic reversion of the *MCM10* exon 14 locus via a break induced replication (BIR)-like mechanism[3]. Reversion may retain (left) or lose (right) the 3' *loxP* site (red triangle) on the functional *MCM10* allele. The direction of BIR is depicted with dashed gray lines and triangles from the upstream (blue) or downstream (green) regions of sequence homology.

Supplementary Fig. 4. *CDC45* and *MCM4* deficiency causes haploinsufficiency but not telomere erosion in HCT116 cell lines.

a) Western blot analyses for MCM10, CDC45 and MCM4, with GAPDH loading controls. Quantification of MCM10, CDC45 and MCM4 levels normalized to loading control, relative to wild-type sample is indicated.

b) Western blot analyses for CDC45 or MCM4 with GAPDH as a loading control. Quantification of CDC45 or MCM4 levels normalized to loading control, relative to wild-type sample is indicated.

c) Quantification of average growth rate in *CDC45*$^{+/-}$ (red) and *MCM4*$^{+/-}$ (orange) cell lines normalized to HCT116 wild type cells. For each cell line $n = 6$; error bars indicate SD and significance was calculated using an unpaired, two-tailed student's *t-test* with *<.05; **<.01, ***<.001. Individual data points are indicated (gray circles).

d) Western blot analyses for chromatin-associated PCNA with Ub-PCNA indicated. Cells were treated without (left) or with 40 J of UV light (right).

e) TRF analysis in HCT116 wild type and *CDC45*$^{+/-}$ cell lines. Estimated PDs and location of peak intensity (yellow dots) are indicated.

f) TRF analysis in HCT116 wild type and *MCM4*$^{+/-}$ cell lines. Estimated PDs and location of peak intensity (yellow dots) are indicated. Source data for panels **a**, **b**, **c**, **d**, **e**, and **f**, including relevant exact p-values, are provided in the Source Data file.

Supplementary Fig. 5. Spontaneous reversion of *MCM10+/-* ST cells rescues t-complex accumulation and proliferation rate.

a) Comparison of average plating efficiency as a measure of clonogenic survival in non-ST and ST cell lines 10 or 12 days after plating HCT116 wild type (blue) and *MCM10*$^{+/-}$ cells (red) related to images in Figure 5g. For quantification *n* = 6. Individual data points are indicated (gray circles). Error bars indicate SD and significance was calculated using a two-tailed student's *t-test* with *<.05; **<.01, ***<.001. Asterisks above each column reflect statistical analysis in comparison to wild type. Statistical analyses between non-ST and ST *MCM10* mutants are also indicated.

b) Measurement of telomere length by DNA combing for completely or partially replicated telomeres related to data in Fig. 7c. The average telomere length for each population is indicated and statistical significance was calculated using two-tailed student's *t-test*. Differences between wild type and mutants are not statistically significant (*n.s.*).

c) Average proliferation rate in HCT116 wild type, *MCM10*$^{+/-}$ and reverted ST cell lines normalized to HCT116 wild type ST3. For each cell line *n* = 5. Individual data points are

indicated (gray circles). Error bars indicate SD and significance was calculated using two-tailed student's *t-test* with *<.05; **<.01, ***<.001.

d) (Top left) Diagram of double-stranded telomere restriction fragment (ds-TRF), telomere circle (t-circle) and telomere complex (t-complex) DNA species from 2D TRF gel electrophoresis. (Right) 2D gel electrophoresis in HCT116 wild type, $MCM10^{+/-}$ and reverted ST cell lines. (Bottom left) Genotyping PCR showing alleles that have one *loxP* site 3' of exon 14 (upper) or a *loxP* scar (lower), as well as exon 14 reverted alleles that have retained or lost the 3' *loxP* site. A faint non-specific band is noted (asterisk). Source data for panels **a**, **b**, **c**, and **d**, including relevant exact p-values, are provided in the Source Data file.

Supplementary table 1. Overlap of novel chromosomal aberrations in HCT116 wild-type and *MCM10+/-* #8 cell lines with common fragile sites (CFSs).

HCT116 expected	CFSs from Mrasek et al., 2010[4]
dup(10)(q24.1q26.3)	No
der(16)t(8;16)(q13;p13.3)	Yes - 8q13 (FRA8F)
der(18)t(17;18)(q21;p11.2)	Yes - 17q21 (FRA17D)
**Additional aberrations in wild-type (PD ~200)**	
inv(16)(p11.2q23)	Yes - 16q23.2 (FRA16D)
der(3)t(3;14)(q27;q11.2)	Yes - 3q27 (FRA3C), 14q11.1 (FRA14D)
**Additional aberrations in *MCM10+/-* #8 (PD ~25)**	
t(5;8)(p15;q13)	Yes - 5p15 (FRA5H); 8q13 (FRA8F)
i(21)(q10)	No
der(1)t(1;10)(q21;q11.2)	Yes - 1q21 (FRA1F); 10q11.2 (FRA10G)
der(10)t(1;10)(q21;q11.2)	Yes - 1q21 (FRA1F); 10q11.2 (FRA10G)
der(5)t(5;10)(p15;q22)	Yes - 5p15 (FRA5H); 10q22.1 (FRAD10D)
der(10)t(5;10)dup(10)	No
del(11)(p11.2)	Yes - 11p11.2 (FRA11L)
del(11)(q23)	Yes - 11q23.3 (FRA11G)
dup(7)(q22q32)	Yes - 7q22 (FRA7F), 7q32.3 (FRA7H)
del(13)(q22q34)	Yes - 13q22 (FRA13E), 13q31 (FRA13H, 13q32 (FRA13D), 13q34 (FRA13I)
del(1)(q32q42)	Yes - 1q32 (FRA1Q), 1q41 (FRA1R), 1q42 (FRA1H)
add(15)(p11.2)	No
**Additional aberrations in *MCM10+/-* #8 (PD ~100)**	
del(6)(q16)	Yes - 6q16.3 (FRA6J)
i(15)(q10)	No
del(2)(q23)	Yes - 2q23 (FRA2S)
add(10)(p11.2)	Yes - 10p11.2 (FRA10J)
del(11)(p11.2)	Yes - 11p11.2 (FRA11L)
add(3)(q21)	Yes - 3q21 (FRA3M)
del(5)(q13)	Yes - 5q13 (FRA5K)
del(11)(q13)	Yes - 11q13.3 (FRA11H)
add(12)(p11.2)	Yes - 12p11.2 (FRA12H)
idic(6)(p23~25)	Yes - 6p23 (FRA6A), 6p25.1 (FRA6B)
del(2)(q31)	Yes - 2q31 (FRA2G)
idic(15)p12	No
del(3)(p11.2)	No
add(19)(p13)	Yes - 19p13.1 (FRA19B)
dic(18;22)(p11.2;p11.2)	No
add(17)(q25p11.2)	Yes - 17q25 (FRA17E), 17p11 (FRA17C)
dic(14;15)(q32;q23~26)	Yes - 14q32 (FRA14H), 15q24 (FRA15), 15q25 (FRA15F), 15q26 (FRA15G)
add(17)(q25)	Yes - 17q25 (FRA17E)
del(13)(q32)	Yes - 13q32 (FRA13D)
t(14;15)(q22;p11)	Yes - 14q22 (FRA14F)
add(20)(p11.2)	Yes - 20p11.23 (FRA20A)
inv(4)(p14;q31)	Yes - 4p14 (FRA4G), 4q31.1 (FRA4C)
der(5)t(5;8)del(5)(q31)	Yes - 5q31.1 (FRA5C)
del(6)(q16)	Yes - 6q16.3 (FRA6J)
**Overlap of *MCM10*$^{+/-}$ aberrations with CFSs**	**80% (28/35)**

List of expected and novel chromosomal aberrations identified in HCT116 wild-type or *MCM10* mutant karyotypes.

Supplementary Table 2. List of all primers and oligos.

MCM10 Locus Specific rAAV targeting F	5'-TTATGTTTCAGGTTCAGGG-3'
MCM10 Locus Specific rAAV targeting R	5'-GGCTAACTTAAGGGAAGAGAGA-3'
MCM10 exon 14 Genotyping F	5'-GGAGGAGTTAGTAAAGCTAGTA-3'
MCM10 exon14 Genotyping R	5'-CTACTGTCAATGCCTCTGGTTAT-3'
MCM10 exon 3 guide oligo	5'-GAAGAAAATAACTTCTTGACG-3'
MCM10 exon 3 Screening F	5'-GGAGACAAGGAGAACAAAGACC-3'
MCM10 exon 3 Screening R	5'-GCTGGCCCAAACATTTCATC-3'
MCM10 exon 3 Sequencing	5'-CCCATTAGGTAGTTTCTCTTACCC-3'
MCM10 exon 3 Illumina Seq F	5'-TCGTCGGCAGCGTCAGATGTGTATAAGAGACAGAAGACAATCTGTCTCTGCTGAC-3'
MCM10 exon 3 Illumina Seq R:	5'-GTCTCGTGGGCTCGGAGATGTGTATAAGAGACAGCCATCATCAGCCTCTTCTGTATAA-3'
CDC45 exon 3 guide oligo	5'-ATACGCTGGTTCCAGTTTCT -3'
CDC45 exon 3 Screening F	5'-TTGTCTCTCAACCCGTCTAATC -3'
CDC45 exon 3 Screening R	5'-TGCTAGATTCCAAGCACCTTAC -3'
CDC45 exon 3 Sequencing	5'-TTGTCTCTCAACCCGTCTAATC -3'
MCM4 exon 2 guide oligo	5'-TTCCACGCCGGCTGCCGCGG -3'
MCM4 exon 2 Screening F	5'-AACGCTAGAGGAACACACCC -3'
MCM4 exon 2 Screening R	5'-GGAATCCTCGCCTCTACGTC -3'
MCM4 exon 2 Sequencing	5'-GCCATTAACTTTCAGGGCCG -3'
MUS81 exon 2 Synthego guide RNA	5'-GGCGCTGCGTTCCCTCCGAC-3'
MUS81 exon 2 Screening F	5'-CTGTCCCAACCCGCTCTTCGTTC -3'
MUS81 exon 2 Screening R	5'-GGATTCGTGGAGTGTGGAGT -3'
MUS81 exon 2 Sequencing	5'-CTGTCCCAACCCGCTCTTCGTTC -3'
TELC oligo (labeled for TRF probe)	5'-CCCTAACCCTAACCCTAACCCTAA-3'

REFERENCES

1 Yeo, G. & Burge, C. B. Maximum entropy modeling of short sequence motifs with applications to RNA splicing signals. *J Comput Biol* **11**, 377-394, doi:10.1089/1066527041410418 (2004).

2 Baxley, R. M., Thu, Y. M. & Bielinsky, A. K. *The Role of Mcm10 in Replication Initiation*. The Initiation of DNA Replication in Eukaryotes edn, 319-341 (Springer International Publishing, 2016).

3 Kramara, J., Osia, B. & Malkova, A. Break-Induced Replication: The Where, The Why, and The How. *Trends Genet* **34**, 518-531, doi:10.1016/j.tig.2018.04.002 (2018).

4 Mrasek, K. *et al.* Global screening and extended nomenclature for 230 aphidicolin-inducible fragile sites, including 61 yet unreported ones. *Int J Oncol* **36**, 929-940 (2010).

Supplemental Figure 1

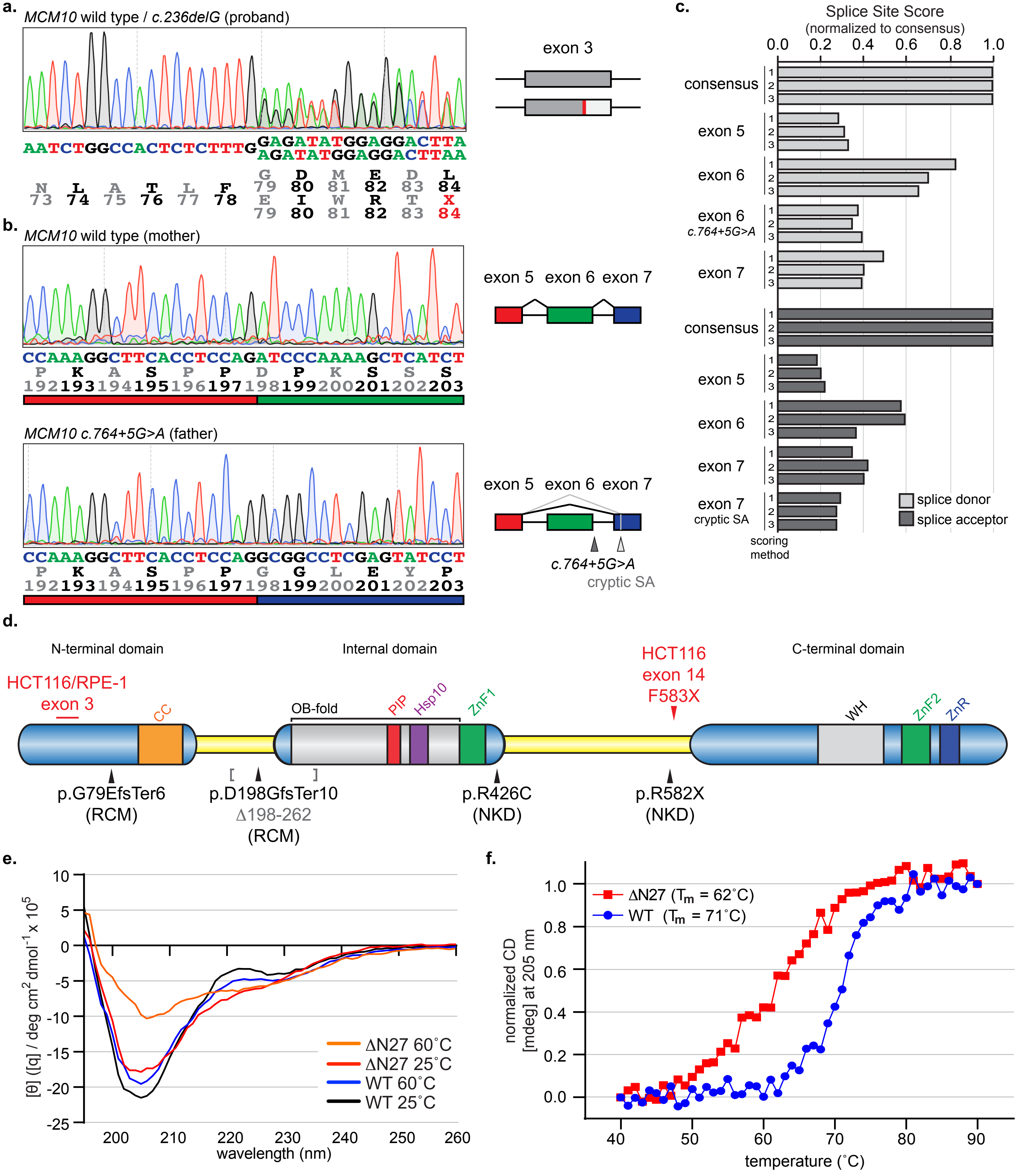

Supplemental Figure 2

a.

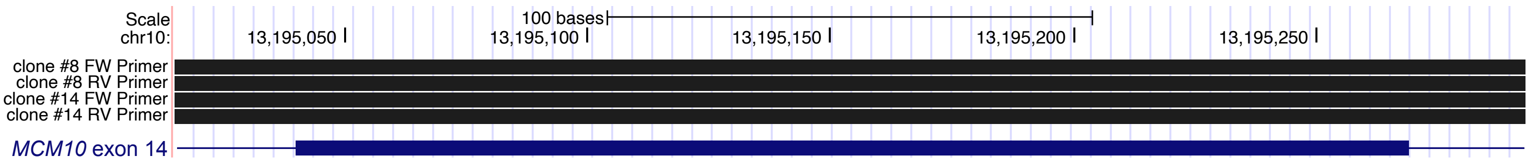

b.

```
HCT116 - MCM10 exon 3
WT           (20bp)-AGTCAGCCTTGGATTGTAATTCAGAAGAAAATAACTTCTTG_ACGCGGGAAAATGGCGAGCCCGACG-(40bp)
#1    -17bp  (20bp)-AGTCAGCCTTGGATTG------AG-----------C-TCTTG_ACGCGGGAAAATGGCGAGCCCGACG-(40bp)
#4    +1bp   (20bp)-AGTCAGCCTTGGATTGTAATTCAGAAGAAAATAACTTCTTGGACGCGGGAAAATGGCGAGCCCGACG-(40bp)
#26   +1bp   (20bp)-AGTCAGCCTTGGATTGTAATTCAGAAGAAAATAACTTCTTGGACGCGGGAAAATGGCGAGCCCGACG-(40bp)

hTERT RPE-1 - MCM10 exon 3
WT           (20bp)-AGTCAGCCTTGGATTGTAATTCAGAAGAAAATAACTTCTTG_ACGCGGGAAAATGGCGAGCCCGACG-(40bp)
#19   -5bp   (20bp)-AGTCAGCCTTGGATTGTAATTCAGAAGAAAATAACTT----_ACGC-GGAAAATGGCGAGCCCGACG-(40bp)
#20   -5bp   (20bp)-AGTCAGCCTTGGATTGTAATTCAGAAGAAAATAACTT----_ACGC-GGAAAATGGCGAGCCCGACG-(40bp)
#24   -2bp   (20bp)-AGTCAGCCTTGGATTGTAATTCAGAAGAAAATAACTTCT--_ACGCGGGAAAATGGCGAGCCCGACG-(40bp)
#29   -2bp   (20bp)-AGTCAGCCTTGGATTGTAATTCAGAAGAAAATAACTTCT--_ACGCGGGAAAATGGCGAGCCCGACG-(40bp)
```

c.

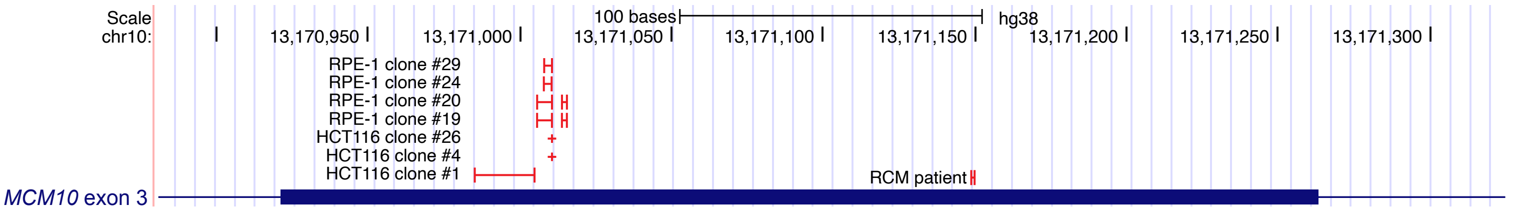

d. Apoptosis Assay Gating Strategy Workflow

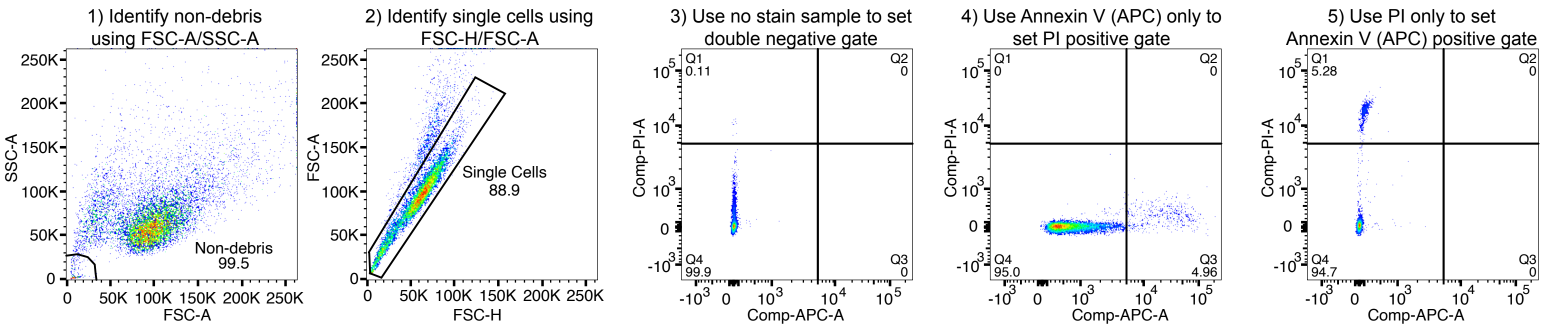

e.

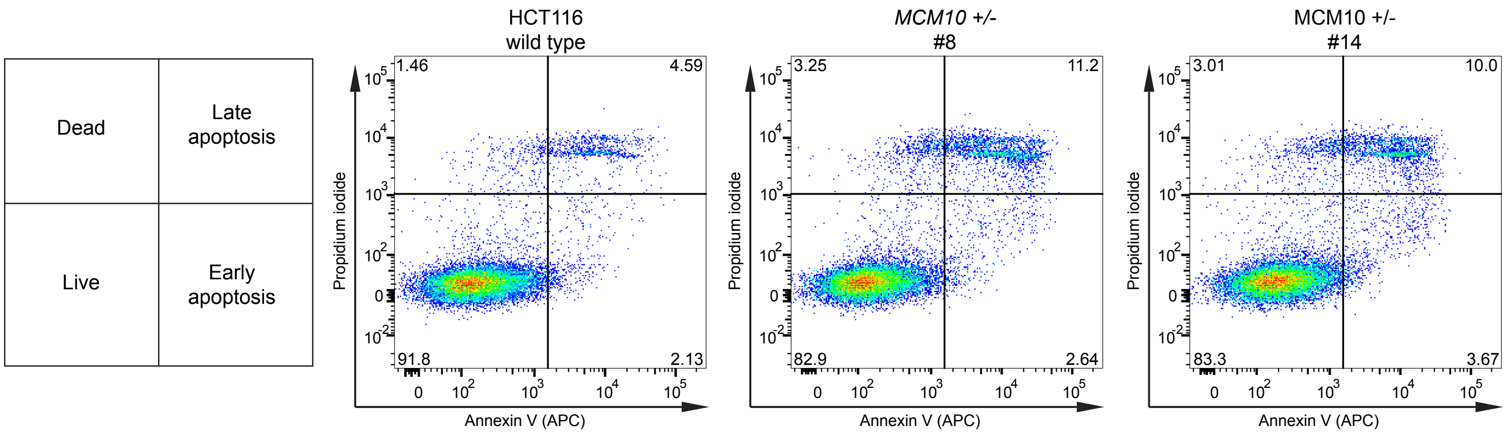

Supplemental Figure 3

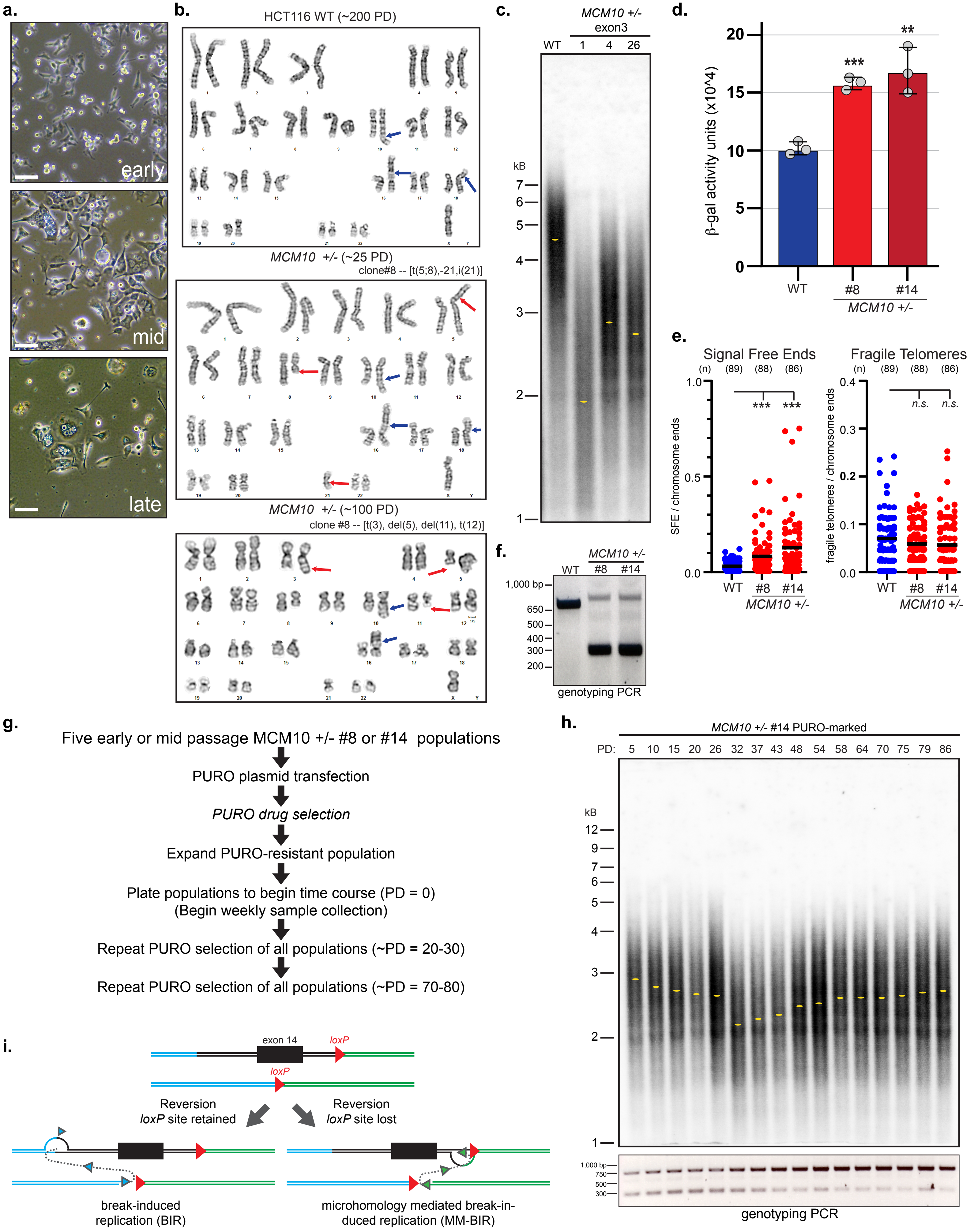

Supplemental Figure 4

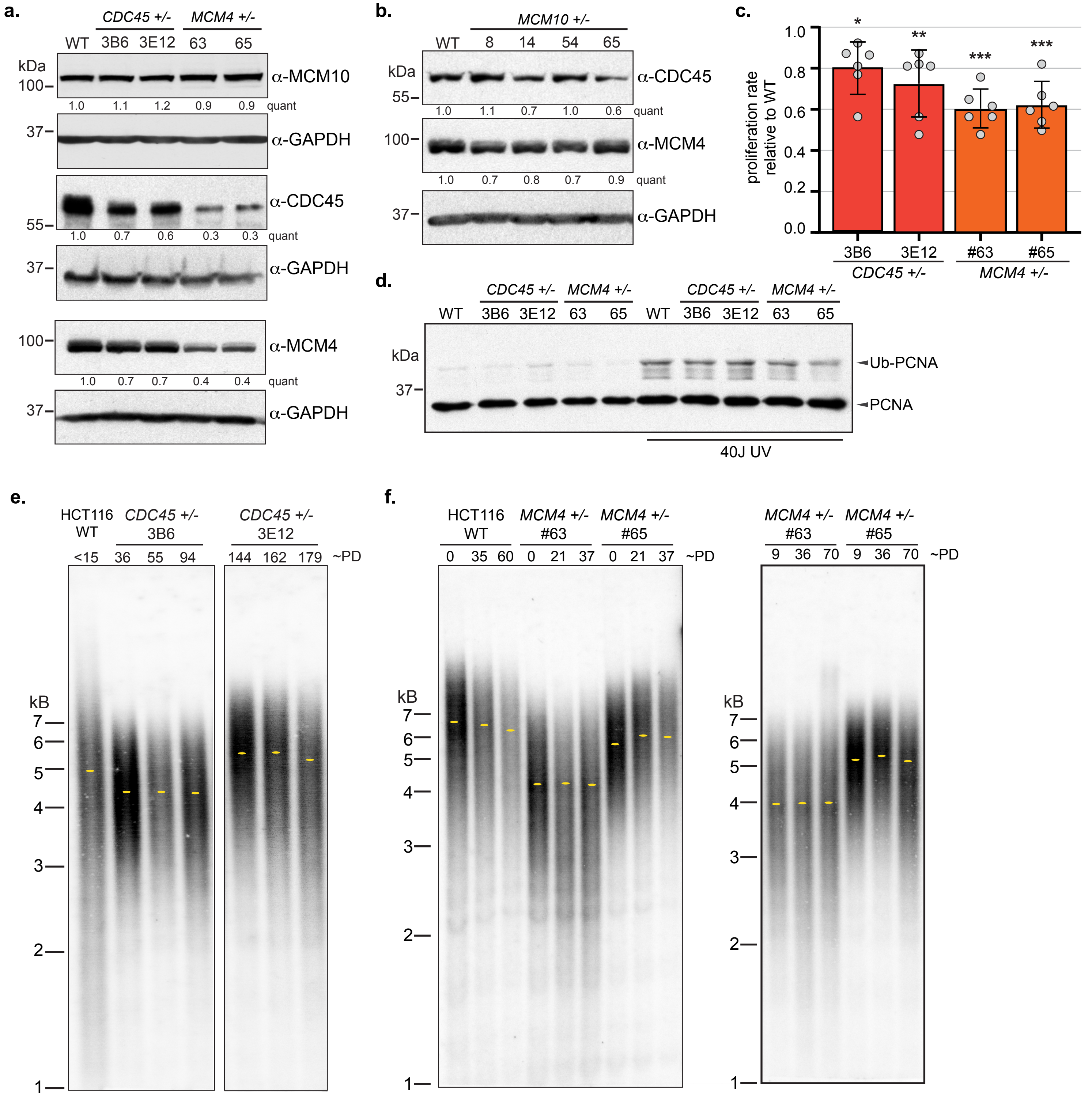

Supplemental Figure 5

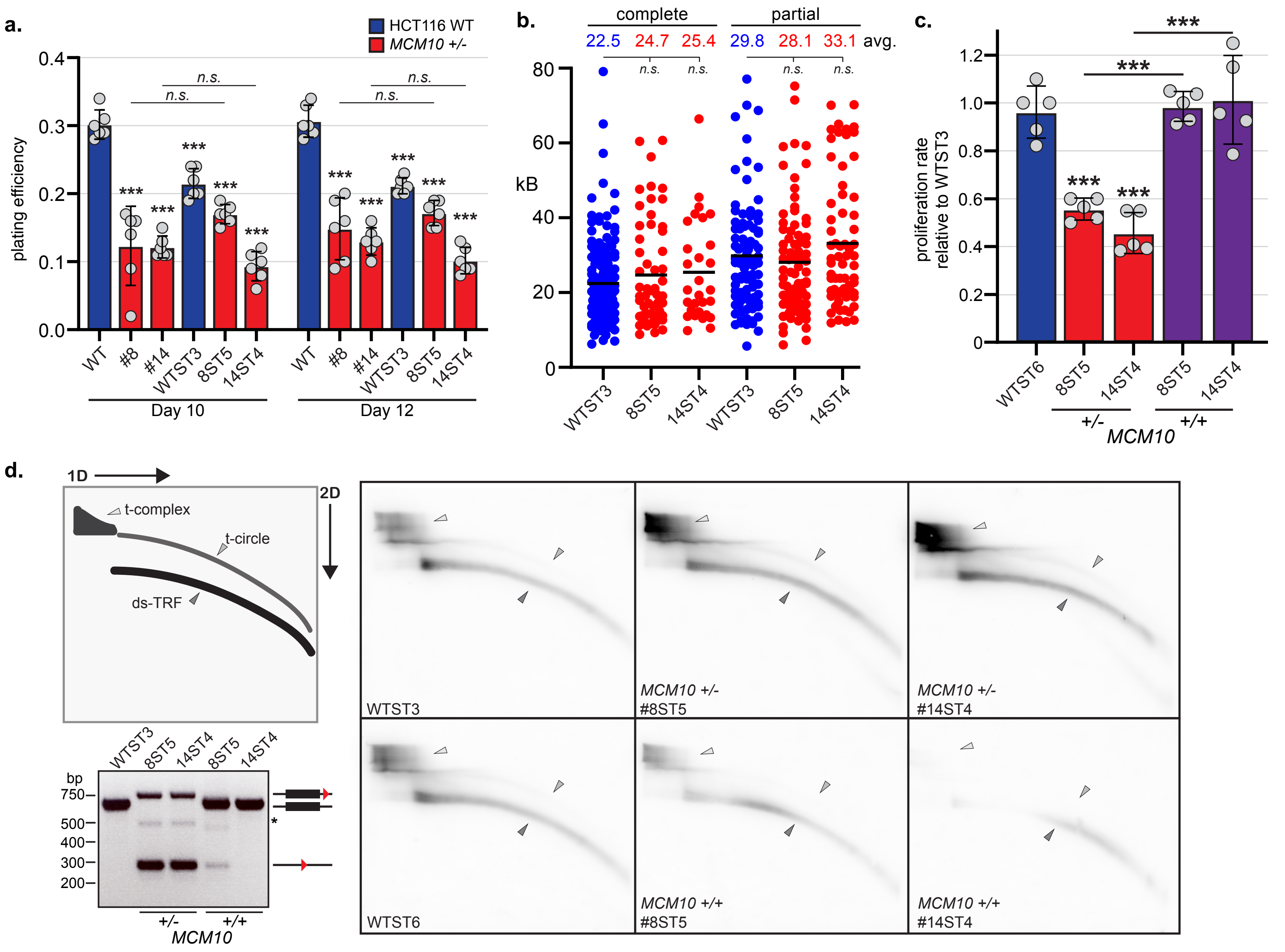

Supplement: Supplementary file 1 — Supplementary Information [file 41467_2021_21878_MOESM1_ESM.pdf]
