# Supplementary material for: Bi-allelic MCM10 variants associated with immune dysfunction and cardiomyopathy cause telomere shortening
Source: Nat Commun. 2021 Mar 12;12:1626. doi: 10.1038/s41467-021-21878-x (PMC7955084; doi:10.1038/s41467-021-21878-x)
Supplement: Supplementary file 3 — Reporting Summary [file 41467_2021_21878_MOESM3_ESM.pdf]

nature research

Corresponding author(s): Anja-Katrin Bielinsky

Last updated by author(s): January 18, 2021

Reporting Summary

Nature Research wishes to improve the reproducibility of the work that we publish. This form provides structure for consistency and transparency in reporting. For further information on Nature Research policies, see our Editorial Policies and the Editorial Policy Checklist.

Statistics

For all statistical analyses, confirm that the following items are present in the figure legend, table legend, main text, or Methods section.

n/a	Confirmed	
☐	☒	The exact sample size (*n*) for each experimental group/condition, given as a discrete number and unit of measurement
☐	☒	A statement on whether measurements were taken from distinct samples or whether the same sample was measured repeatedly
☐	☒	The statistical test(s) used AND whether they are one- or two-sided<br>*Only common tests should be described solely by name; describe more complex techniques in the Methods section.*
☒	☐	A description of all covariates tested
☒	☐	A description of any assumptions or corrections, such as tests of normality and adjustment for multiple comparisons
☐	☒	A full description of the statistical parameters including central tendency (e.g. means) or other basic estimates (e.g. regression coefficient) AND variation (e.g. standard deviation) or associated estimates of uncertainty (e.g. confidence intervals)
☐	☒	For null hypothesis testing, the test statistic (e.g. *F*, *t*, *r*) with confidence intervals, effect sizes, degrees of freedom and *P* value noted<br>*Give P values as exact values whenever suitable.*
☒	☐	For Bayesian analysis, information on the choice of priors and Markov chain Monte Carlo settings
☒	☐	For hierarchical and complex designs, identification of the appropriate level for tests and full reporting of outcomes
☒	☐	Estimates of effect sizes (e.g. Cohen's *d*, Pearson's *r*), indicating how they were calculated

Our web collection on statistics for biologists contains articles on many of the points above.

Software and code

Policy information about availability of computer code

Data collection	All software used for data collection is commercially available or open source, including: Platypus version 0.5.2; VariantStudio version 2.2 (Illumina); Ingenuity VA (Qiagen); Nexus Discovery Edition (BioDiscovery, Inc., El Segundo, CA); MaxEntScan (Yeo and Burge, 2003); FIJI version 2.1.0/153.c (https://imagej.net/Fiji); FCS Express version 6 (De Novo Software); FlowJo version 10.6.1; Genomic Vision EasyScan service (https://store.genomicvision.com/services/77-easyscan.html); Genomic Vision FiberStudio version 2.0.
Data analysis	All software used for data analysis is commercially available or open source, including: Platypus version 0.5.2; VariantStudio version 2.2 (Illumina); Stampy version 1.0.20 (Lunter and Goodson, 2011); Ingenuity VA (Qiagen); Nexus Discovery Edition (BioDiscovery, Inc., El Segundo, CA); MaxEntScan (Yeo and Burge, 2003); Tracking of Indels by Decomposition version 3.2.0 (TIDE; https://tide.nki.nl); FIJI version 2.1.0/153.c (https://imagej.net/Fiji); Microsoft Excel version 16.44; FCS Express version 6 (De Novo Software); FlowJo version 10.6.1; Genomic Vision FiberStudio version 2.0; Adobe Photoshop version 21.2.1; GraphPad Prism version 8.2.0, RStudio version 1.1.442.

For manuscripts utilizing custom algorithms or software that are central to the research but not yet described in published literature, software must be made available to editors and reviewers. We strongly encourage code deposition in a community repository (e.g. GitHub). See the Nature Research guidelines for submitting code & software for further information.

Data

Policy information about availability of data

All manuscripts must include a data availability statement. This statement should provide the following information, where applicable:

- Accession codes, unique identifiers, or web links for publicly available datasets
- A list of figures that have associated raw data
- A description of any restrictions on data availability

The authors declare that all data related to the findings of this study are available within the article and supplemental information or are available from the

corresponding author upon reasonable request. The Protein Data Bank accession codes for the Xenopus laevis MCM10-ID are 3EBE16 [https://www.ncbi.nlm.nih.gov/Structure/pdb/3EBE] and 3H1574 [https://www.ncbi.nlm.nih.gov/Structure/pdb/3H15]).

Field-specific reporting

Please select the one below that is the best fit for your research. If you are not sure, read the appropriate sections before making your selection.

☒ Life sciences ☐ Behavioural & social sciences ☐ Ecological, evolutionary & environmental sciences

For a reference copy of the document with all sections, see nature.com/documents/nr-reporting-summary-flat.pdf

Life sciences study design

All studies must disclose on these points even when the disclosure is negative.

Sample size	We did not perform calculations to determine sample size. Sample size for experiments was chosen based on standards for studies of human cell lines (for example Thakar et. al., PMID: 32358495; Harvey et. al., PMID: 30410680) attempting to have at least three biologically independent replicates and reproducibility sufficient to accurately test for statistical significance. Whenever possible analyses were carried out on two or more completely independent, clonal cell lines, further validating experimental conclusions. All cell lines were maintained in appropriate numbers and confluency required to ensure healthy cultures, and experiments were performed on sufficiently low-passage cultures unless late-passage cell lines were specifically desired.
Data exclusions	No data were excluded.
Replication	Experimental findings were reproduced in a minimum of two biological replicates and typically in multiple independent, clonal cell lines. All results were reproducible. The only exceptions were the "+ telomerase inhibitor" samples on the TRF gel in Figure 6b. This represents the only reverted MCM10 mutant cell line that arose during growth in telomerase inhibitor. We continued to propagate in the presence of telomerase inhibitor for 67 population doublings and analyzed 9 separate timepoints, all of which showed a reproducible phenotype that was consistent with all other analyses in the paper.
Randomization	For molecular DNA combing and t-FISH experiments samples were analyzed randomly and quantified events were randomly sampled on each slide or coverslip. Randomization was not applicable to other assays performed here.
Blinding	All molecular DNA combing and t-FISH experiments were imaged and analyzed in a blinded manner. For other assays blinding is less feasible and involve experimental measurement of objective parameters, and are therefore not affected when analyzed in an unblinded manner.

Reporting for specific materials, systems and methods

We require information from authors about some types of materials, experimental systems and methods used in many studies. Here, indicate whether each material, system or method listed is relevant to your study. If you are not sure if a list item applies to your research, read the appropriate section before selecting a response.

Materials & experimental systems

n/a	Involved in the study	
☐	☒	Antibodies
☐	☒	Eukaryotic cell lines
☒	☐	Palaeontology and archaeology
☒	☐	Animals and other organisms
☐	☒	Human research participants
☒	☐	Clinical data
☒	☐	Dual use research of concern

Methods

n/a	Involved in the study	
☒	☐	ChIP-seq
☐	☒	Flow cytometry
☒	☐	MRI-based neuroimaging

Antibodies

Antibodies used	All antibody dilutions are included in the Methods section of the manuscript.<br><br>Primary antibodies include anti-MCM10 (Bethyl Laboratories A300-131A); anti-MCM10 (Novus H00055388-D01P); anti-CDC45 (Santa Cruz sc-55568); anti-MCM4 (Santa Cruz sc-28317); anti-MUS81 (Abcam ab14387); anti-PCNA (Abcam ab290; anti-Ubiquityl-PCNA (Lys164; Cell Signaling Technology mAb13439, D5C7P) anti-RPA32 (S4/8; Bethyl Laboratories A300-245A); anti-GAPDH (GeneTex GTX627408); anti-alpha-Tubulin (Millipore Clone DM1A, T9026); anti-Lamin B1 (Proteintech #12987); anti-MCM2 (BD Biosciences 610700); anti-ssDNA (Immuno-Biological Laboratories 18731); anti-BrdU (clone B44, BD Biosciences 347580); anti-BrdU (U1/75(ICR1), Abcam ab6326).<br><br>Secondary antibodies inlcude anti-Mouse HRP (Jackson Laboratories 115-035-003); anti-Rabbit HRP (Jackson Laboratories 111-035-144); anti-Mouse HRP (BioRad 1706516); anti-Rabbit HRP (GE Healthcare NA9340); anti-Mouse AF488 (Jackson

	ImmunoResearch 715-545-140); anti-Mouse Cy3.5 (Abcam ab6946); anti-Rat Cy5 (Abcam ab6565); anti-Rabbit BV480 (BD Horizon 564879); anti-Rat AF555 (Invitrogen A21434).
Validation	All antibodies used in this study are commercially available.<br>> anti-MCM10 (Bethyl Laboratories A300-131A). Reactivity: human. Applications: WB, IP. Validated against cell lysates from HeLa, HEK293T and Jurkat cells.<br>> anti-MCM10 (Novus H00055388-D01P). Reactivity: human. Applications: WB. Validated against cell lysates from transfected 293T cells.<br>> anti-CDC45 (Santa Cruz sc-55568). Reactivity: mouse, rat, human. Applications: WB, IP, IF, ELISA. Validated against cell lysates from non-transfected 293T and Jurkat, and transfected 293T cells.<br>> anti-MCM4 (Santa Cruz sc-28317). Reactivity: mouse, rat, human. Applications: WB, IP, IF, IHC(P), ELISA. Validated against nuclear extracts from HeLA, NIH/3T3 and KNRK, and cell lysates from K-562 and L8 cells.<br>> anti-MUS81 (Abcam ab14387). Reactivity: human, not mouse. Applications: WB, Flow Cyt. Validated against cell lysates from HeLa cells.<br>> anti-PCNA (Abcam ab29). Reactivity: mouse, rat, chicken, human, Drosophila melanogaster, monkey, Zebrafish. Applications: WB, IHC(P), ICC/IF, Flow Cyt. Validated against cell lysates from HeLa, PC12, SV40LT-SMC and NIH 3T3 cell lines, and lysates from Rat liver and heart.<br>> anti-Ubiquityl-PCNA (Lys164; Cell Signaling Technology mAb13439, D5C7P). Reactivity: human. Applications: WB, IP. Validated against cell lysates from untreated or UV-treated HeLa cells.<br>> anti-RPA32 (S4/8; Bethyl Laboratories A300-245A). Reactivity: mouse, human. Applications: WB, IP, IHC, ICC/IF. Validated against cell lysates from untreated or etoposide treated HeLa cells, and immunoprecipitates from untreated, etoposide, or etoposide/Lamda phosphatase treated HeLa cells.<br>> anti-GAPDH (GeneTex GTX627408). Reactivity: human, mouse, rat, Zebrafish, yeast, Drosophila, pig, monkey, Candida albicans, E. coli. Applications: WB, ICC/IF, ICH(P), EMSA. Validated against cell lysate from Drosophila, yeast, 293T, NIH 3T3 and PC-12 cells, and brain lysate from mouse and rat.<br>> anti-alpha-Tubulin (Millipore Clone DM1A, T9026). Reactivity: rat, mouse, pig, bovine, avian, guinea pig, human. Applications: WB, IC, IF. Validated against cell lysate from A431 cells.<br>> anti-Lamin B1 (Proteintech #12987). Reactivity: (tested) human, mouse, rat; (cited) canine, chicken, hamster, human, mouse, rat, swine. Applications: WB, IP, IHC, IF, FC, CHIP, ELISA. Validated against cell lysates from HeLa, HepG2 and Jurkat cells.<br>> anti-MCM2 (BD Biosciences 610700). Reactivity: human, mouse, rat, dog, chicken. Applications: WB, IF, IP. Validated against cell lysate from human endothelial cells.<br>> anti-ssDNA (Immuno-Biological Laboratories 18731). Reactivity: single-stranded DNA of all species, including human. Applications: IHC. Validated against ssDNA in Naruse et. al., Histochemsitry, 1994.<br>> anti-BrdU (clone B44, BD Biosciences 347580). Reactivity: Iodouridine, Bromouridine. Applications: IHC, Flow Cyt. Validated via Flow Cyt or IHC of BrdU labeled cells.<br>> anti-BrdU (U1/75(ICR1), Abcam ab6326). Reactivity: Chlorouridine, Bromouridine. Applications: ICC/IF, IHC(P), Flow Cyt. Validated via Flow Cyt or ICC/IF of BrdU labeled HeLa cells or IHC(P) of BrdU labeled rat small intestine.

Eukaryotic cell lines

Policy information about cell lines

Cell line source(s)	HCT116 and hTERT RPE-1 cell lines were obtained from ATCC.
Authentication	Cell lines used in this study were authenticated using several methods including: morphology check by microscope, growth curve analyses, PCR and Sanger sequencing, and high-quality G-band karyotype analyses.
Mycoplasma contamination	All cell lines used in this study tested negative for mycoplasma contamination.
Commonly misidentified lines (See ICLAC register)	No commonly misidentified cell lines were used in this study.

Human research participants

Policy information about studies involving human research participants

Population characteristics	The "population" reported in this manuscript consists of a single family that included three male fetuses diagnosed with restrictive cardiomyopathy with lymphoreticular hypoplasia.
Recruitment	The "participants" included the three affected male fetuses, the parents, and three unaffected siblings. Each "participant" was included in an effort to identify a genetic cause for the observed pathology in the affected fetuses. Following the identification of causative, compound heterozygous mutations in MCM10, all further efforts focused on modeling these mutations in human cell lines. Therefore, no additional human research participants were recruited in relation to our study.
Ethics oversight	Patients participated under the Molecular Genetic Analysis and Clinical studies of Individuals and Families at Risk of Genetic Disease (MGAC) protocol approved by West Midlands Research Ethics Committee, reference number 13/WM/0466.

Note that full information on the approval of the study protocol must also be provided in the manuscript.

Flow Cytometry

Plots

Confirm that:

☒ The axis labels state the marker and fluorochrome used (e.g. CD4-FITC).

☒ The axis scales are clearly visible. Include numbers along axes only for bottom left plot of group (a 'group' is an analysis of identical markers).

☒ All plots are contour plots with outliers or pseudocolor plots.

☒ A numerical value for number of cells or percentage (with statistics) is provided.

Methodology

Sample preparation	Flow cytometry analyses of cell cycle, DNA synthesis and origin licensing, was performed on HCT116 and hTERT RPE-1 cell lines. Briefly, cells were incubated with 10 µM EdU (Santa Cruz sc-284628) for 30 min before harvesting with trypsin. Soluble proteins were extracted in CSK (10 mM PIPES pH 7.0, 300 mM sucrose, 100 mM NaCl, 3 mM MgCl2 hexahydrate) with 0.5% Triton X-100, then cells were fixed in PBS with 4% PFA (Electron Microscopy Services) for 15 min. Cells were labeled with 1 µM AF647-azide (Life Technologies A10277) in 100 mM ascorbic acid, 1 mM CuSO4, and PBS to detect EdU for 30 min, at room temperature. Cells were washed, then incubated with MCM2 antibody 1:200 (BD Biosciences #610700) in 1% BSA in PBS with 0.5% NP-40 for 1 hr at 37°C. Next, cells were washed and labeled with donkey anti-mouse AF488 secondary antibody 1:1,000 (Jackson Immunoresearch 715-545-150) for 1 hr at 37°C. Lastly, cells were washed and incubated in DAPI (Life Technologies D1306) and 100 ng/mL RNase A (Sigma R6513) overnight at 4°C.<br><br>Flow cytometry analysis of apoptosis was performed on HCT116 and hTERT RPE-1 cells. Cells were seeded in 6-well plates and allowed to proliferate for approximately 72 hr. Adherent and floating cells were collected, washed with 1x PBS twice, and stained using the APC Annexin V apoptosis detection kit (Biolegend 640932) according to the manufacturer's instructions.
Instrument	Attune NxT (Beckman Coulter), LSR II (BD Biosciences) or FACSCanto A V0730042 (BD Biosciences).
Software	FCS Express 6 (De Novo Software), FlowJo v10.6.1 and Microsoft Excel.
Cell population abundance	All flow cytometry experiments identified specific cell populations based on the gating strategy explained previously. Cells were not sorted and utilized for downstream experiments, therefore the purity of populations was not determined.
Gating strategy	For flow cytometry analyses of cell cycle FSC-A/SSC-A was used to resolve the main population from debris. 405-H/405-A was used to isolate single cells. Non-EdU labeled or non-primary antibody samples were used to determine EdU-positive or MCM-positive gates, respectively. The gating strategy for this technique has been previously published and is available in Matson et al., 2017, Figure 1 - figure supplement 1 (DOI: 10.7554/eLife.49040).<br><br>For flow cytometry analysis of apoptosis FSC-A/SSC-A was used to resolve the main population from debris. FSC-H/FSC-A was used to isolate single cells. No-PI or No-APC stained samples were used to determine PI-positive or APC-positive gates, respectively. The gating strategy for this technique has been included in Supplemental Figure 2d.

☒ Tick this box to confirm that a figure exemplifying the gating strategy is provided in the Supplementary Information.
